# Chemotherapy accelerates immune-senescence and functional impairments of Vδ2^pos^ T cells in elderly patients affected by liver metastatic colorectal cancer

**DOI:** 10.1186/s40425-019-0825-4

**Published:** 2019-12-11

**Authors:** Elena Bruni, Valentina Cazzetta, Matteo Donadon, Matteo Cimino, Guido Torzilli, Gianmarco Spata, Gloria Leonardi, Francesco Dieli, Joanna Mikulak, Domenico Mavilio

**Affiliations:** 1 Unit of Clinical and Experimental Immunology, Humanitas Clinical and Research Center – IRCCS, Via Alessandro Manzoni, 56, Rozzano, Milan, Italy; 2 Department of Medical Biotechnologies and Translational Medicine (BioMeTra), University of Milan, Milan, Italy; 3 Department of Hepatobiliary and General Surgery, Humanitas University, Humanitas Clinical and Research Center – IRCCS, Rozzano, Milan, Italy; 4 Central Laboratory for Advanced Diagnosis and Biomedical Research, Palermo, Italy; 5 Department of Biomedicine, Neurosciences and Advances Diagnostics (Bi.N.D.), University of Palermo, Palermo, Italy

**Keywords:** γδ T cells, Immune-senescence/Aging, Cancer, Chemotherapy

## Abstract

Human (gamma delta) γδ T cells are unconventional innate-like lymphocytes displaying a broad array of anti-tumor activities with promising perspectives in cancer immunotherapy. In this context, Vδ2^pos^ T cells represent the preferential target of several immunotherapy protocols against solid tumors. However, the impact of both aging and chemotherapy (CHT) on Vδ2^pos^ T cells is still unknown. The present study evaluates with multi-parametric flow cytometry the frequencies, terminal differentiation, senescence and effector-functions of peripheral blood and tumor infiltrating Vδ2^pos^ T cells purified from liver metastases (CLM) of patients affected by colorectal cancer (CRC) compared to those of sex- and age-matched healthy donors. The peripheral blood of CLM patients underwent CHT is characterized by decreased amounts of Vδ2^pos^ T cells showing a relative increase of terminally-differentiated CD27^neg^/CD45RA^pos^ (T_EMRA_) cells. The enrichment of this latter subset is associated with an increased expression of the senescent marker CD57. The acquisition of CD57 on T_EMRA_ Vδ2^pos^ T cells is also coupled with impairments in cytotoxicity and production of TNF-α and IFN-γ. These features resemble the acquisition of an immune-senescent profile by Vδ2^pos^ T cells from CLM patients that received CHT, a phenomenon that is also associated with the loss of the co-stimulatory marker CD28 and with the induced expression of CD16. The group of CLM patients underwent CHT and older than 60 years old showed higher frequencies of CD57^pos^ and T_EMRA_ Vδ2^pos^ T cells. Similar results were found for tumor infiltrating Vδ2^pos^ T cell subset purified from CLM specimens of patients treated with CHT. The toxicity of CHT regimens also affects the homeostasis of Vδ2^pos^ T cells by inducing higher frequencies of circulating CD57^pos^ T_EMRA_ subset in CLM underwent CHT and younger than 60 years old. Taken together, our data demonstrate that the enrichment of senescent Vδ2^pos^ T cells in CLM patients is not only induced by patients’ aging but also by the toxicity of CHT that further accelerates the accumulation of CD57^pos^ T_EMRA_ cells highly dysfunctional in their anti-tumor activities. These results are important to both predict the clinical outcome of CLM and to optimize those protocols of cell cancer immunotherapy employing unconventional Vδ2^pos^ T cells.

## Introduction

Human γδ T lymphocytes are divided in the two main Vδ1^pos^ and Vδ2^pos^ subsets on the basis of their TCRδ-chain repertoire. While Vδ1^pos^ cells preferentially localize in mucosal tissues and skin, Vδ2^pos^ T cells are mainly enriched in peripheral blood (PB) where they represent about 5% of all circulating T cells. The activation of Vδ2^pos^ T cells relies on the recognition of non-peptidic compounds (i.e. microbial or stress- or tumor-induced “phosphoantigens”) in association with butyrophilin 3A1 (BTN3A1 also known as CD277). Besides the TCR interactions with phosphoantigens/BTN3A1 complexes, several Natural Killer Receptors (NKRs) are involved in triggering the anti-tumor functions of Vδ2^pos^ T cells, with the C-lectin type NKG2D playing a major role [1, 2]. The differential expressions of CD27 and CD45RA surface markers identify different Vδ2^pos^ T cell subsets: CD27^pos^/CD45RA^pos^ naïve cells (T_Naïve_), CD27^pos^/CD45RA^neg^ central memory (T_CM_), CD27^neg^/CD45RA^neg^ effector-memory (T_EM_) and the terminally-differentiated (T_EMRA_) CD27^neg^/CD45RA^pos^ cells. These Vδ2^pos^ T cell subsets diverge not only for their maturation/differentiation status, but also for proliferative capacities, effector functions and resistance to cell death in response to antigens and/or cytokine stimulations [3].

Growing evidences highlighted the high impact of Vδ2^pos^ T cells in cancer immune-surveillance with promising perspectives in cancer immunotherapy [4, 5]. In this context, two main clinical approaches have been employed to boost anti-tumor activities of Vδ2^pos^ T cells. The first one activates them through the in vivo administration of either IL-2 or synthetic nitrogen-containing bisphosphonates (NBPs) drugs that, in turn, induce intracellular accumulation of phosphoantigens. A second strategy relies on adoptive transfers of Vδ2^pos^ T cells expanded in vitro with several methodologies such as the activation with zoledronate [5, 6]. However, these procedures showed both experimental and clinical limits and many efforts are currently being implemented to further improve the effector-functions and persistence in vivo of Vδ2^pos^ T cells. In this context, cellular senescence is certainly one of the main issues to solve considering that age-related changes of T cells greatly impair their capacity to expand and proliferate, thus leading to dysfunctional immune responses against tumors and pathogens [7]. The shift to senescence and accumulation of mature T cells physiologically occur after 60 years old when both αβ and γδ T lymphocytes lose their co-stimulatory molecules (i.e. CD27 and CD28), acquire terminally-differentiated T_EM_ and T_EMRA_ phenotypic profiles, express high constitutive levels of the senescence marker CD57 and shorten their telomerase lengths [8–11]. However, it is still controversial whether CD57 can be used as a single marker to identify senescent Vδ2^pos^ T cells regardless of differential expression of CD27 and CD45 [3, 11, 12].

Aging is certainly a major burden for social health systems in the industrialized countries as the populations are longer exposed to several pro-tumorigenic risk factors. This leads to a significant higher incidence of cancer onsets in the 6th, 7th and 8th decades of life [13]. Hence, there are rising numbers of elderly patients undergoing anti-cancer conventional chemotherapies (CHT), whose high toxicities greatly hamper both duration and quality of life. In this regard, several lines of clinical and experimental evidence pointed out that these anti-neoplastic treatments further accelerate immune-cell senescence, thus representing negative prognostic factors in aging and worsening the overall clinical outcomes of cancer patients [14, 15].

Since the use of Vδ2^pos^ T cells is currently considered one of the most promising tools in cancer immunotherapy [4, 5], understanding the exact impact of CHT on their immune-senescence is key to better predict the clinical outcomes of cancer in elderly and to optimize those therapeutic protocols targeting these highly cytotoxic unconventional T cell effectors. Colorectal cancer (CRC) represents the 3rd most frequent solid cancer and more than 50% of CRC patients undergo hepatic dissemination of the primary tumor. The gold-standard therapeutic approach of CRC patients with liver metastasis (CLM) is the surgical removal of hepatic secondary lesions after neoadjuvant combination CHT with or without biological therapy (BT) (Table 1) [16, 17]. Moreover, a higher infiltration of competent immune cells in tumor mass greatly improves the prognosis of CLM patients and increases their overall survival (OS) [18, 19]. Here, we analyze the impact of conventional CHT regimens on the homeostasis and effector-functions of Vδ2^pos^ T cells in a cohort of CLM elderly patients.

**Table 1:** Neoadjuvant combination chemotherapy (CHT) with or without biological therapy (BT) of enrolled CLM patients

	Patients (number)	Patients (%)	CHT cycles (mean number ± SD)
CHT/BT Regimens^a^	58	82	8.7 ± 5.3
Combination Therapy with Biologicals			
FOLFOX + VEGF-A mAb	12	21.5	7.7 ± 1.4
FOLFIRI + EGFR mAb	11	19.0	11.7 ± 4.3
FOLFIRI + VEGF-A mAb	10	17.2	7.5 ± 3.3
FOLFIRI + FOLFOX + VEGF-A mAb	7	12.0	13.0 ± 3.2
FOLFOX + EGFR mAb	6	10.3	11.0 ± 2.3
XELOX + VEGF-A mAb	4	6.9	8.5 ± 3.4
Combination Therapy without Biologicals			
FOLFOX	4	6.9	5.0 ± 1.6
XELOX	2	3.4	4.6 ± 1.2
FOLFIRI	2	3.4	7.0 ± 6.0
Naïve for CHT	13	18	0.0
Total Patients	71		

FOLFOX: 5-fluorouracil/oxaliplatin; XELOX: capecitabine/oxaliplatin; FOLFIRI: 5-fluorouracil/irinotecan

*EGFR mAb* Epidermal Growth Factor Receptor inhibitor monoclonal antibody

*VEGF-A mAb* Vascular Endothelial Growth Factor A monoclonal antibody

^a^Note:

a) All CLM patients completed their last CHT cycle at least 6 weeks before the blood draws used for our experiments and before surgical procedures

b) The table refers all therapies received by CLM patients before surgery

c) More than 91% of all CLM patients received one line therapy and all other patients received two lines (1^st^ and 2^nd^) combination therapy: 3 patients received 1^st^ FOLFOX and 2^nd^ FOLFIRI + VEGF-A; 1 patient received 1^st^ FOLFIRI + VEGF-A and 2^nd^ FOLFOX + VEGF-A, and 1 patient received 1^st^ FOLFIRI + VEGF-A and 2^nd^ FOLFOX

## Methods

### Patients and specimen collections

Biological specimens from CLM patients underwent CHT (*n = 58*), or from CHT naïve patients (*n = 13*) and aged- and sex-matched healthy donors (*n = 40*) (Table 1). Patients’ recruitment was performed according to the Declaration of Helsinki and the protocol had been approved by the Institutional Review Board (IRB) of Humanitas Research Hospital (HRH) (Approval N.168/18). All enrolled patients signed the related consent forms. Liver specimens and peripheral blood mononuclear cells (PBMCs) were isolated and stored as we previously described [19, 20].

### Flow cytometry

Absolute γδ T cell counts were performed on 100 μl of fresh PB stained with following anti-human monoclonal antibodies (mAbs): CD3 (SK7; BV605) and CD45 (H130; AF700) (BioLegend) and Vδ2 (IMMU-389; FITC) (Beckman Coulter). We then used CountBright™ Absolute Counting Beads (Invitrogen) according to the manufacturer’s instructions.

For both regular and intracellular staining, γδ T cells were first screened for viability with Zombie Aqua™ Fixable Viability kit (BioLegend) and then processed as previously described [20]. The following mAbs were used: CD28 (CD28.2; PE-Cy7) (BioLegend); Vδ2 (B6; BUV395), CD3 (UCHT1; BUV661), CD45RA (H100; BUV737), CD16 (348; BUV496) (BD); CD57 (REA769; PE-Vio615) (Miltenyi); CD27 (0322; APCeFluor780) (eBioscience). The intracellular amounts of TNF-α (Mab11; PE) and IFN-γ (B27; Bv711) (BD) as well as the frequency of cytotoxic CD107a^pos^ cells (H4A3, PE) (BD Biosciences) was evaluated after stimulating γδ T cells with Phorbol myristate acetate (PMA; 0.5 μg/mL) and Ionomycin (0.1 μg/mL) (Sigma Aldrich).

Flow cytometry experiments were performed on FACS Symphony™ (BD). All data and *t-SNE * algorithm were analyzed with FlowJo Software (version 9.6) (FlowJo LLC) using single stained controls BD CompBeads™ (BD).

### Statistical analyses

The data were assessed by non-parametric *Mann-Whitney U* (unpaired) or *Wilcoxon* (matched-paired) tests by using *GraphPad Prism* version 7. For all correlation analysis Pearson’s coefficient was applied. Statistically significant *p* values were represented with GraphPad (GP) style and summarized with following number of asterisks (*): *P ≤0.05; **P ≤0.01; ***P ≤0.001; ****P ≤0.0001.

## Results

**Fig. 1:**
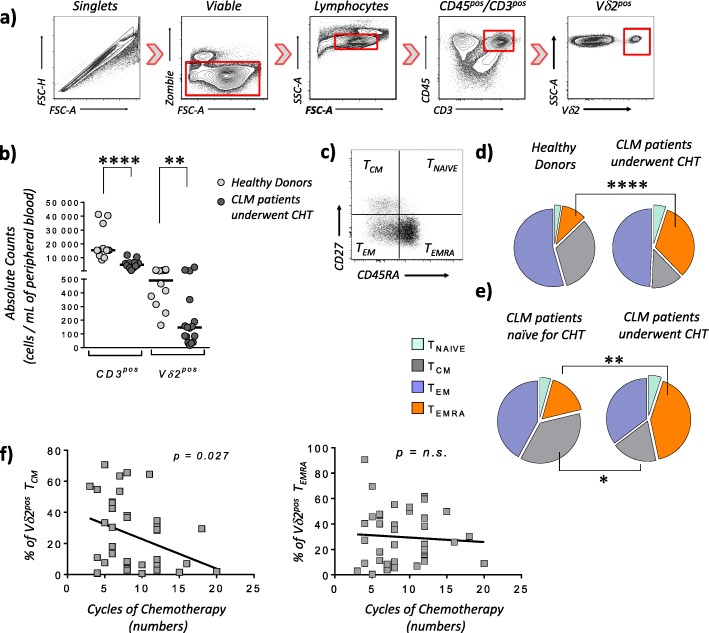
Frequency and distributions of peripheral blood Vδ2^pos^ T cell subsets in patients affected by liver metastasis of colorectal cancer and underwent chemotherapy. **a** Representative dot plot flow cytometric graphs showing the gating strategy of viable CD45^pos^/CD3^pos^/Vδ2^pos^ T lymphocytes. **b** Statistical dot plot graph showing the absolute number of CD3^pos^ (left) and Vδ2^pos^ (right) T cells per 1 mL of blood in healthy donors (*n = 12; mean age: 49.3 ± 9.5*) and CLM patients underwent CHT regimens (*n = 16; mean age: 51.5 ± 8.1*). **c**-**e** Representative dot plot graph flow cytometric graph (**c**) and pie charts (**d** and **e**) showing respectively the distribution and the percentages of CD27^pos^/CD45RA^pos^ T_Naive_ (upper right in dot plot graph and light green in pie charts), CD27^pos^/CD45RA^neg^ central memory (T_CM_) (upper left in dot plot graph and gray in pie charts), CD27^neg^/CD45RA^neg^ effector-memory (T_EM_) (lower left in dot plot graph and purple in pie charts) and terminally-differentiated CD27^neg^/CD45RA^pos^ (T_EMRA_) (lower right in dot plot graph and orange in pie charts) Vδ2^pos^ T cell subsets. Pie charts compare the frequencies of Vδ2^pos^ T cell subsets between healthy donors (*n = 34; mean age: 51.7 ± 10.8*) with age-matched CLM patient underwent CHT (*n = 33; mean age: 51.5 ± 8.1*) **d** as well as between CLM patients naïve for CHT (*n = 13; mean age: 69.5 ± 8.1*) and age-matched CLM patients underwent CHT (*n = 41; mean age: 70.1 ± 6.5*) (**e**). **f** Statistical analysis showing the Pearson correlations between the frequency (%) of either T_CM_ (left) or T_EMRA_ (right) Vδ2^pos^ T cells with the number of CHT cycles (*mean number: 8.7 ± 6.5*) administered to patients affected by CLM (*n = 40*)

Vδ2^pos^ T cells were gated within viable CD3^pos^/CD45^pos^ lymphocytes and their absolute counts are significantly lower in the PB of CLM patients underwent CHT compared to those of healthy donors (Fig. 1a-b). We then analyzed the surface expression of CD27 and CD45RA to track the differentiation and distribution of Vδ2^pos^ T cell subsets. Our data showed a significant increase of Vδ2^pos^ T_EMRA_ in CLM patients underwent CHT (28.9 ± 20.6%) compared to healthy controls (9.4 ± 6.4%). This phenomenon is associated with the previous administration of CHT, as the frequency of circulating Vδ2^pos^ T_EMRA_ in those CLM patients naïve for CHT (16.7% ±12.6) is similar to that of healthy donors and significantly lower to that of CLM patients underwent CHT (41.6% ±19.6). The increased amounts of Vδ2^pos^ T_EMRA_ in CLM patients treated with CHT is counterbalanced by a significant decrease of Vδ2^pos^ T_CM_ in the same patients compared to their counterparts naïve for CHT (Fig. 1c-d-e). The great impact of neoadjuvant CHT in shaping the distribution of Vδ2^pos^ T cell subsets in CLM patients is also confirmed by our findings showing that the number of CHT cycles (8.7 ± 2.7) inversely correlates with the percentages of PB Vδ2^pos^ T_CM_, while not affecting at all the overall frequencies of PB Vδ2^pos^ T_EMRA_ (Fig. 1f). This latter dichotomy reflects the different homeostatic status of Vδ2^pos^ T_CM_ compared to that of Vδ2^pos^ T_EMRA_, as the first subset is composed of proliferating lymphocytes high susceptible to the toxicity of those chemotherapy compounds that kills all dividing cells without any specificity against tumor blasts. Instead, T_EMRA_ Vδ2^pos^ cells are terminally differentiated and not proliferating effectors resistant to CHT, thus explaining their high frequency even after several cycles of neoadjuvant anti-tumor chemotherapies.

**Fig. 2:**
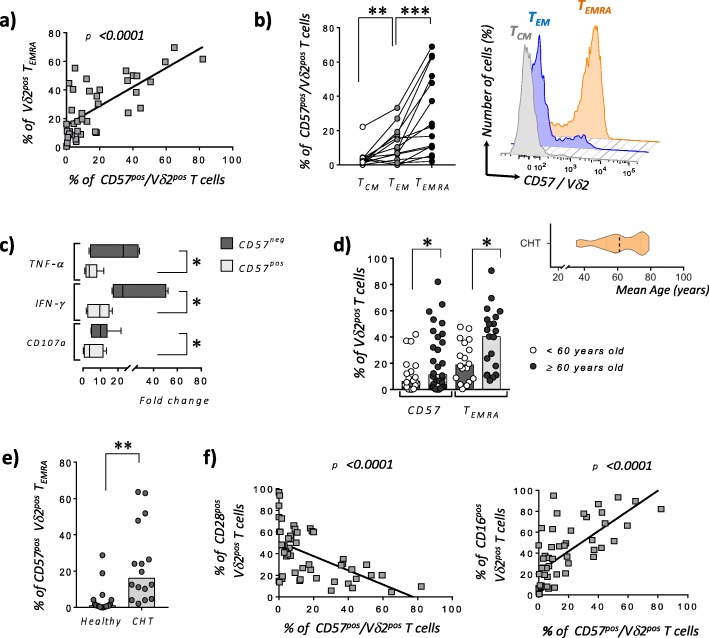
Senescence of peripheral blood Vδ2^pos^ T cell in patients affected by liver metastasis of colorectal cancer and underwent chemotherapy. **a** Statistical analysis showing the correlations between the frequencies (%) of Vδ2^pos^ T_EMRA_ and CD57^pos^/Vδ2^pos^ T and in CLM patients underwent CHT (*n = 40*). **b** Statistical dot plot (left) and representative histogram (right) graphs showing the expressions (%) of CD57 on matching T_CM_, T_EM_ and T_EMRA_ Vδ2^pos^ T cell subsets in CLM patients underwent CHT (*n = 15*). **c** Statistical bar graphs showing the fold change increases of CD107a expression as well as of intracellular amounts of IFN-γ and TNF-α by CD57^neg^ and CD57^pos^ Vδ2 T cell effector subsets (i.e. T_EMRA_ and T_EM_) from CLM patients underwent CHT and following in vitro stimulation with PMA and Ionomycin (*n = 6*). **d** Statistical dot plot analysis showing the expressions (%) of CD57 and the frequencies (%) of T_EMRA_ within Vδ2^pos^ T cell compartments in CLM patients underwent CHT and divided in two groups of respectively < (white circles; *n = 18)* and ≥ (black circles; *n = 21*) of 60 years old. The mean age of the entire cohort of CLM patients underwent CHT is of 61 ± 10.7 years old as shown in statistical graph on right upper side. **e** Statistical dot plot analysis showing the expressions (%) of CD57 on Vδ2^pos^ T_EMRA_ cells from CLM patients underwent CHT and under 60 years old (*n = 16*) compared to age-matched healthy donors (*n = 16*). **f** Statistical analysis showing the correlations between the surface levels (%) of CD57 and CD28 (*n = 51*) (left graph) or CD16 (*n = 51*) (right graph) on Vδ2^pos^ T cells in CLM patients underwent CHT

The relative increased frequency of PB T_EMRA_ Vδ2^pos^ in CLM patients underwent CHT correlates with their higher expression of CD57. Notably, the expression of this latter marker of immune senescence follows the terminal differentiation of Vδ2^pos^ T cells. Indeed, the frequency of PB CD57^pos^ T_EMRA_ Vδ2^pos^ T cells resulted significantly higher compared to that of CD57^pos^ T_EM_ Vδ2^pos^ T cells that, in turn, showed significantly higher amounts of CD57 when compared to T_CM_ Vδ2^pos^ T cells (Fig. 2a-b). The acquisition of CD57 by terminal-differentiated Vδ2^pos^ T cells is also associated with significantly impaired effector-functions in term of anti-tumor cytokines production (i.e. IFN-γ and TNF-α) and ability to degranulate (i.e. decreased amounts of cytotoxic CD107a^pos^ cells) when compared to CD57^neg^/Vδ2^pos^ T cells (Fig. 2c). Taken together, these data indicate that the PB of CLM patients underwent CHT is highly enriched of senescent CD57^pos^/ T_EMRA_ Vδ2^pos^ T cells dysfunctional in their anti-tumor effector functions.

**Fig. 3:**
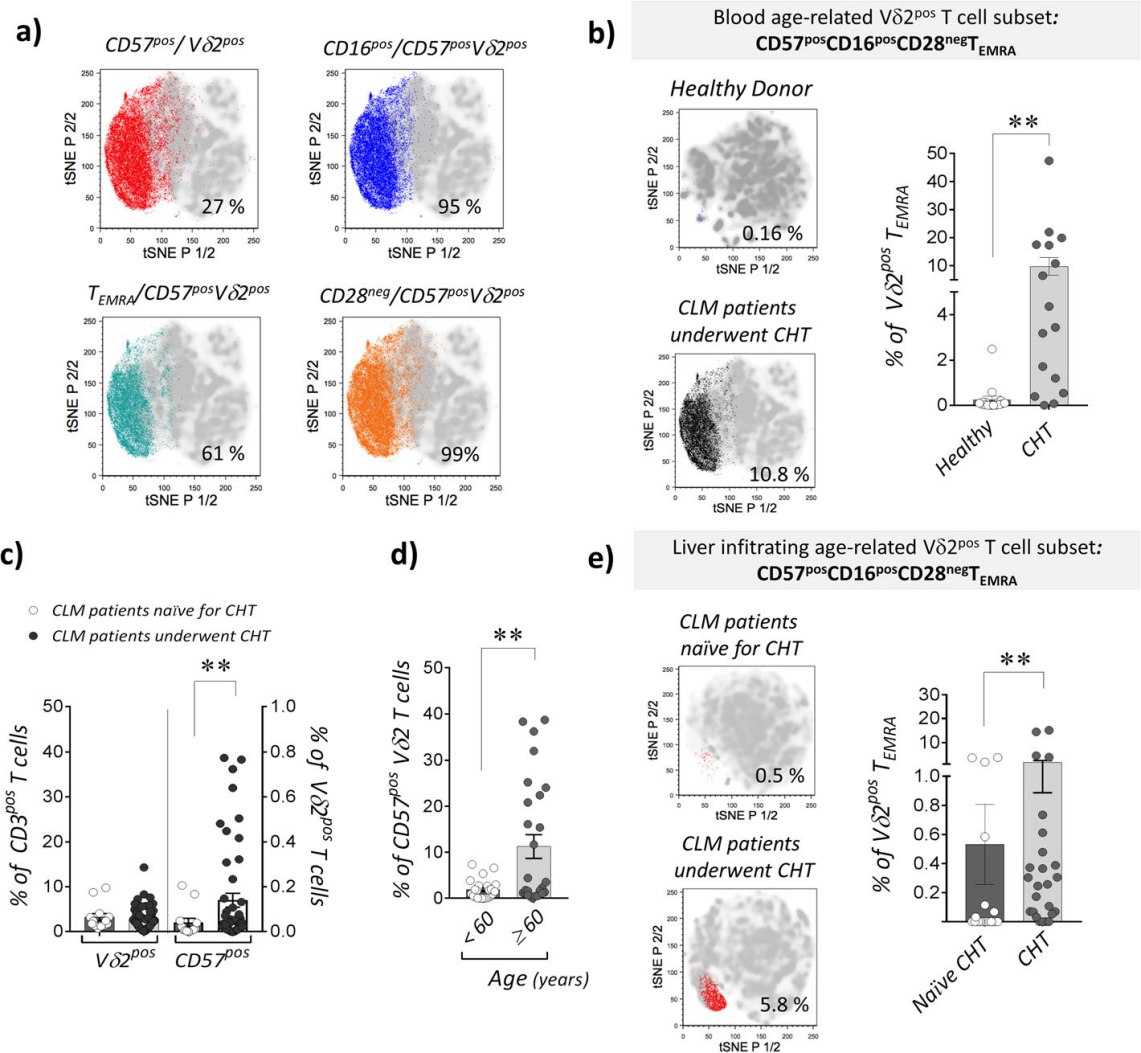
Clustering of peripheral blood and tissue infiltrating senescent CD57^pos^/CD28^neg^/CD16^pos^ T_EMRA_ Vδ2^pos^ T cells in patients affected by liver metastasis of colorectal cancer and underwent chemotherapy. **a** *t-SNE* analysis plots in CLM patients underwent CHT (*n = 16*) showing the cluster of PB CD57^pos^/ Vδ2^pos^ T cells (red, upper left plot) co-expressing CD16 (blue, upper right plot), CD45RA but not CD27 (T_EMRA_ in green, lower left plot) and negative for CD28 (black, lower right plot). **b** t-SNE analysis plots (left) and statistical dot plot graph (right) showing the frequency (%) of senescent PB CD57^pos^/CD28^neg^/CD16^pos^ T_EMRA_ Vδ2^pos^ T cells in healthy donors (upper plot; *n* = 12; mean age: 51.7 ± 10.8) and CLM underwent CHT (lower plot; *n = 16; mean age: 61 ± 10.7*). **c** Summary dot plot analysis showing the frequencies (%) of liver tumor-associated Vδ2^pos^ T cells within total CD3^pos^ T lymphocytes or CD57^pos^/Vδ2^pos^ T cells in CLM patients receiving CHT regimen (black circles; *n* = 58) and naïve for CHT (white circles; *n* = 13). **d** Statistical dot plot analysis showing the frequencies (%) of CD57^pos^ cells on liver tumor infiltrating Vδ2^pos^ T cells in CLM patients underwent CHT regimen and sub-divided in two groups of respectively < (white circles; *n* = 22) and ≥ (black circles; (*n* *= 27*) of 60 years old. **e** t-SNE analysis plots (left plots) and statistical chart (right graph) of the CHT-mediated changes in the frequency (%) of the age-related, liver tumor infiltrating CD57^pos^CD28^neg^CD16^pos^T_EMRA_ Vδ2^pos^ T cell cluster in CHT treated CLM patients (lower plot, *n = 25; mean age: 61 ± 10.7*) and naïve for CHT patients (upper plot, *n* *= 13*; *mean age: 69.5 ± 8.1*)

To assess the impact of patients’ aging in the higher frequencies of CD57^pos^ and T_EMRA_ Vδ2^pos^ T cells in CLM patients underwent CHT, we divided this cohort in subjects younger or older than 60 years old. Our data confirmed that the age-induced immune-senescence significantly increases the percentages of both CD57^pos^ and T_EMRA_ Vδ2^pos^ T cells in those patients > 60 years old (Fig. 2d). We also showed that CHT alone induces immune-senescence regardless of patients’ age. Indeed, the percentage of CD57^pos^ T_EMRA_ Vδ2^pos^ cells resulted significantly higher in those CLM underwent CHT and younger than 60 years old compared to that of age-matched healthy donors (Fig. 2e). These data clearly indicate that both CHT and aging play synergic roles in the regulation of Vδ2^pos^ T cell homeostasis in CLM patients with the final result of greatly accelerating their terminal differentiation towards a senescent CD57^pos^/T_EMRA_ subset highly impaired in its anti-tumor effector-functions. We also demonstrate here that the acquisition of CD57 inversely correlates with the surface expression of CD28 while being associated with increased surface amounts of CD16 (Fig. 2f), the FcγRIII receptor known to define highly differentiated human Vδ2^pos^ T_EMRA_ cells [21]. The clustering of CD57^pos^/Vδ2^pos^ T_EMRA_ co-expressing CD16 and lacking CD28 in CLM patients underwent CHT is confirmed and better visualized by the t-Distributed Stochastic Neighbor Embedding (*t-SNE*) analysis (Fig. 3a). This analytic approach also allowed us to compare the impact of CHT in inducing high frequencies of PB CD57^pos^/CD16^pos^/CD28^neg^/Vδ2^pos^ T_EMRA_ cells in CLM patients compared to those of age-matched healthy donors (Fig. 3b).

Although the overall frequency of tumor infiltrating Vδ2^pos^ T cells purified from CLM specimens is not affected by the administration of CHT, we found a significant increase of CD57 expression on those Vδ2^pos^ T cells from patients underwent CHT compared to naïve ones (Fig. 3c). Similar to their PB counterparts, the frequency of CD57^pos^/Vδ2^pos^ T cells is significantly higher in elderly CHT patients ≥60 years old (Fig. 3d). Consistently with these data, t-SNE analysis showed also in CLM specimens of patient administered with CHT an increased frequency of age-related tumor infiltrating CD57^pos^/CD28^neg^/CD16^pos^ T_EMRA_ Vδ2^pos^ T lymphocytes (Fig. 3e).

## Discussion

The present study is aimed to measure the true impact of conventional CHT regimens on unconventional T cell senescence in elderly cancer patients, since the toxicity of conventional anti-tumor therapies greatly impairs their ability to clearance malignant cells [7, 12, 14, 15]. In particular, we focused our investigations on circulating Vδ2^pos^ cells that are endowed with great anti-tumor potentials currently being targeted by several protocols of cancer immunotherapies [4–6]. Our data showed that CLM patients underwent CHT, although showing lower absolute counts of circulating Vδ2^pos^ cells, retain high relative frequencies of terminally differentiated and senescent CD57^pos^/CD28^neg^/CD16^pos^ T_EMRA_ Vδ2^pos^ cells greatly impaired in their effector-functions. This latter subset is resistant to the toxicity exerted by repeated CHT cycles administering DNA-damaging drugs that, in contrast, are highly toxic against less differentiated and still proliferating T_CM_ Vδ2^pos^ cells.

The preferential accumulation in PB of senescent CD57^pos^ T_EMRA_ Vδ2^pos^ cells in CLM patients underwent CHT is associated with two major mechanisms. The first one is linked to natural immune-senescence of people aging as the incidence of many cancers is higher in patients ≥ of 60 years old. In this context, liver metastatic CRC is one of the most common causes of cancer deaths worldwide with a higher incidence in elderly [16, 17]. Indeed, our cohort of recruited CLM subjects had a mean age of 61 ± 10.7 years old and both the frequencies of CD57^pos^ and T_EMRA_ Vδ2 T cell subsets resulted higher in that fraction of patients older than 60 years. The second mechanism is associated with a direct toxicity of CHT on immune cells, as also highlighted by several studies both in pediatric and geriatric cancer patients [14, 15, 22]. As a matter of fact, we show here that the expression of CD57 on T_EMRA_ Vδ2^pos^ cells is much higher on those CLM patients underwent CHT and younger than 60 years old compared to age-matched healthy donors. This demonstrates that neoadjuvant CHT induces immune senescence also on unconventional T cells regardless of CLM patients’ age. Notably, high frequencies of impaired CD57^pos^/T_EMRA_ Vδ2^pos^ cells were able to persist in PB of CLM patients even after 6 weeks from the completion of the last CHT cycle and before surgical removal of liver metastases. Further prospective studies are required to assess how long senescent and functional impaired Vδ2^pos^ T cells survive after CHT and what clinical impact they have on the OS of CLM patients. In this regard, it has been already reported that the enrichment of circulating subsets of CD57^pos^ αβ T cells represents a negative prognostic factor in the clinical outcome of gastrointestinal cancers [23].

Our study also contributes to better characterize immune-senescence of Vδ2^pos^ T cells, since it has been recently reported that expression of CD57 can define alone their senescent status without the need of also evaluating the expression of both CD27 and CD45RA [11]. This represents a key point that is currently being debated both in physiological and pathological conditions. We found that, at least in a human cancer setting, the expression of CD57 on senescent Vδ2^pos^ T cell parallels their terminal differentiation towards T_EMRA_ (CD27^neg^/CD45RA^pos^), a phenomenon associated with the loss of CD28 and the acquired expression of CD16. These results are in line with a previous study showing that Vδ2^pos^ T_EMRA_ are refractory to phosphoantigen stimulation, but rather respond to activation via FcγRIII [21].

The majority of cancer patients are older than 65 years old in line with population aging [14]. In this context, several clinical trials in the elderly are currently being implemented to optimize the anti-tumor activities of unconventional T cells. These therapeutic protocols are mostly aimed to expand Vδ2^pos^ T cells both in vivo and in vitro [6]. Hence, a better understanding of the mechanisms accelerating immune-senescence in aging is fundamental to boost the effector-functions of these cytotoxic and cytokine-producer T lymphocytes. We show here that, neoadjuvant CHT regimens, although absolutely required to reduce tumor mass in CLM patients before surgery, greatly speed the senescence of Vδ2^pos^ T cells in synergy with aging of cancer patients. This knowledge will allow us to better optimize immune-therapies against cancers in elderly. Indeed, senescence process can be reversed through the inhibition of p38 mitogen-activated protein kinase (MAPK) signaling [24]. This methodology could be then approached to develop new protocols implementing pre-treatment with MAPK inhibitors in elderly patients with CRC [25]. Alternatively, new methodology can be implemented in vitro to select and expand CD57^neg^/Vδ2^pos^ T cells that better resist to the terminal differentiations and senescence induced by CHT. Further studies are also required to better identify those CHT associated accumulation of impaired and senescent circulating Vδ2^pos^ T cells.

## Data Availability

The dataset generated and analyzed in the current study are available from the corresponding authors on reasonable request.

## Abbreviations

CHT: Chemotherapy
CLM: Colorectal liver metastatic cancer
CRC: Colorectal cancer
NBPs: Nitrogen-containing bisphosphonates
T_CM_: Central memory T
T_EM_: Effector memory T
T_EMRA_: Terminally differentiated T
T_Naïve_: Naïve T
*t-SNE*: t-Distributed Stochastic Neighbor Embedding
γδ T: Gamma delta T
